# The utilization of hypoalbuminemia as a prognostic metric in patients with spinal metastases: A scoping review

**DOI:** 10.1016/j.bas.2025.104223

**Published:** 2025-02-25

**Authors:** Jessica Ryvlin, Namal Seneviratne, Ali Haider Bangash, C. Rory Goodwin, Michael H. Weber, Raphaële Charest-Morin, John H. Shin, Anne L. Versteeg, Mitchell S. Fourman, Saikiran G. Murthy, Yaroslav Gelfand, Reza Yassari, Rafael De la Garza Ramos

**Affiliations:** a Spine Tumor Mechanics and Outcomes Research (TUMOR) Lab, Montefiore Medical Center, Albert Einstein College of Medicine, Bronx, NY, USA; b Department of Neurology, Montefiore Medical Center, Albert Einstein College of Medicine, Bronx, NY, USA; c Department of Neurosurgery, Montefiore Medical Center, Albert Einstein College of Medicine, Bronx, NY, USA; d Department of Neurosurgery, Duke University School of Medicine, Durham, NC, USA; e Department of Orthopedic Surgery, University of Connecticut, Farmington, CT, USA; f Department of Orthopedic Surgery, University of British Columbia, Vancouver, BC, Canada; g Department of Neurosurgery, Massachusetts General Hospital, Harvard Medical School, Boston, MA, USA; h Division of Surgery, Department of Orthopaedics, University of Toronto, Toronto, Canada; i Department of Orthopedic Surgery, Montefiore Medical Center, Albert Einstein College of Medicine, Bronx, NY, USA

**Keywords:** Albumin, Spine metastasis, Metastatic spine disease, Spine oncology, Survival, Complications

## Abstract

**Introduction:**

Hypoalbuminemia is associated with poor outcomes in cancer patients, but its role in spinal metastases remains unclear.

**Research question:**

This study aimed to identify albumin cutoff values defining hypoalbuminemia and describe the association between serum albumin and outcomes in patients with spinal metastases.

**Material and methods:**

A narrative review of articles up to December 2022 was conducted using PubMed/Medline, EMBASE, and Web of Science databases. Variables extracted included study design, patient characteristics, serum albumin levels, treatments, and levels of evidence. Outcomes included survival/mortality, complications, ambulatory status, readmission, length of stay, discharge disposition, and blood loss.

**Results:**

Thirty-eight studies comprising 21,401 patients were analyzed. Most studies (92%) were Level of Evidence III. Albumin was evaluated as a continuous variable in 18% of studies and as a dichotomous variable in 76%, with 3.5 g/dL being the most common threshold for hypoalbuminemia. Primary outcomes evaluated were survival/mortality (71% of studies), complications (34%), and reoperation/readmission (11%). Of studies examining the association between hypoalbuminemia and survival/mortality, 74% found a significant association. An association between albumin levels and complications was found in 54% of relevant studies.

**Discussion and conclusion:**

The findings of this study suggest that a threshold of 3.5 g/dL seems most appropriate to define hypoalbuminemia in patients with spinal metastases. However, evidence also supports a level-dependent effect. The most consistent significant association was between low albumin and survival at both fixed and continuous time points. There is less evidence to support an association between hypoalbuminemia and other endpoints such as perioperative complications.

## Highlights

• Hypoalbuminemia in spinal metastases patients is most commonly defined as <3.5 g/dL.
• Low albumin is consistently associated with decreased survival in spinal metastases.
• Evidence for the association of hypoalbuminemia with complications is less consistent.
• The effect of hypoalbuminemia on outcomes appears to be level-dependent.
• Further research is needed on the role of hypoalbuminemia in post-treatment morbidity in spinal metastatic disease patients.

## 1 Introduction

Hypoalbuminemia can be encountered in illness and has been associated with malnutrition and chronic inflammatory states (Gatta et al., 2012; Soeters et al., 2019). Cytokine-mediated peripheral albumin degradation and decreased oral intake leading to relative protein deficiency have both been specifically attributed to patients with advanced cancer, in which hypoalbuminemia has been explored as a clinical biomarker for many years (Gatta et al., 2012; Kim et al., 2017; Suzuki et al., 2013). Studies describing independent associations between hypoalbuminemia and poor outcomes such as length of hospital stay, postoperative complications, and mortality have been conducted in a variety of primary tumor types, including those of the colon, ovary, and nasopharynx, among others (Liao et al., 2021; Fang et al., 2021; Yang et al., 2022; Kengsakul et al., 2022). More recently, application of this prognostic metric to spinal oncology patients has been undertaken, with a significant body of literature published in the past two decades describing the association between hypoalbuminemia and outcomes.

Oncologic surgery for metastatic spinal tumors is known to be associated with significant risk of morbidity and mortality given the inherent frailty of advanced cancer patients along with the complex and often invasive surgical approaches (Luksanapruksa et al., 2017a). Short-term and long-term outcomes in this population have been shown to be influenced by factors such as preoperative functional status, primary tumor type, frailty, muscle mass, and albumin levels, among others (Luksanapruksa et al., 2017b; Paulino et al., 2016; Bakhsheshian et al., 2022; Hersh et al., 2022; Zakaria et al., 2020; De et al., 2022). Regarding albumin, cut-off values defining clinically relevant hypoalbuminemia remain inconsistent in the literature and the multifactorial nature of low serum albumin has brought into question whether this condition is a potentially modifiable risk factor.

Understanding the role of hypoalbuminemia-related inflammation and malnutrition in patients with spinal metastases may guide medical optimization practices and oncologic treatment courses. As such, our objective was to conduct a scoping review of the literature to 1) identify albumin level cutoff values used to define hypoalbuminemia in patients with metastatic spine disease, and to 2) describe the association between serum albumin level and operative/non-operative outcomes.

## 2 Methods

The Preferred Reporting Items for Systematic Reviews and Meta-Analyses (PRISMA) guidelines were followed in this study (Liberati et al., 2009). Institutional review board approval was not needed given the lack of identifiable patient information. A comprehensive online search was done utilizing the Pubmed/Medline, Embase, and Web of Science databases for publications up until December 2022. No language limitation was used. Our initial search terms were derived from the National Cancer Institute’s Nutrition in Cancer Care website – “albumin”, “malnutrition”, “weight loss”, “albumin”, “sarcopenia”, “muscle mass”, “cachexia”, “anorexia”, “visceral fat”, “subcutaneous fat” and “spinal metastasis”.

Studies were included if they were original full-length manuscripts that met the following criteria: 1) patients with spinal metastases, 2) albumin levels evaluated as either continuous or dichotomous values with a cutoff value defining hypoalbuminemia, 3) association with clinical outcomes including survival/mortality, postoperative complications, wound complications, reoperation, readmission, ambulation, functional status, discharge disposition, blood loss, and others. Studies were excluded if they were not full-length original manuscripts (i.e. abstracts, case reports, commentaries, editorials, or reviews). Performance assessment and external validation studies were additionally excluded if they included assessment of albumin using previously established models.

**Fig. 1:**
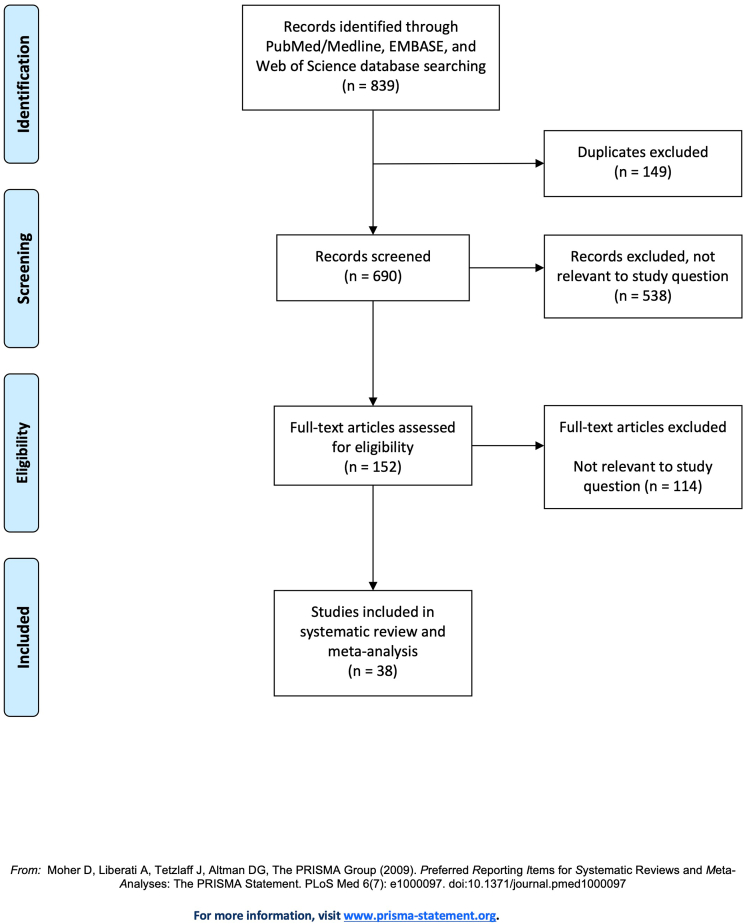
Study selection algorithm.

After removal of duplicates, articles were screened for eligibility based on title and abstract. Full texts were then reviewed, and potential disagreements were resolved amongst the following authors: RDLGR, JHS, CRG, RCM, and MHW. The complete study selection algorithm is depicted in Fig. 1. Data collected from each study included Level of Evidence as defined by the North American Spine Society (NASS), study design, sample size, patient demographic makeup, albumin level/cutoff value defining hypoalbuminemia, treatment type, studied outcomes, and results.

Our primary objective was to identify albumin level cutoff values used to define hypoalbuminemia in patients with metastatic spine disease. Our secondary objective was to and describe the association between serum albumin level and outcomes including survival, complications, readmission/reoperation, ambulatory and functional status, discharge disposition, and others.

A narrative review was performed along with review of descriptive statistics. A meta-analysis was not done given the heterogeneity in studied outcomes.

## 3 Results

### 3.1 Overall findings

After removal of duplicates and articles meeting exclusion criteria, a total of 38 original studies published between 1998 and 2022 were included in our final analysis (Yang et al., 2022; De et al., 2022; Boaro et al., 2020; Bongers et al., 2022; Cook and Baker, 2020; DiSilvestro et al., 2021; Dohzono et al., 2019; Ehresman et al., 2021; Gelfand et al., 2021; Ghori et al., 2015; Guo et al., 2003; Han et al., 2016; Hersh et al., 2018; Hussain et al., 2019; Karhade et al., 2016, 2019a, 2019b, 2022; Kato et al., 2016; Kumar et al., 2015; Liu et al., 2018; Lun et al., 2019; McPhee et al., 1998; Molho et al., 2022; Ogihara et al., 2006; Oh et al., 2012; Paulino et al., 2019; Pennington et al., 2021a, 2021b; Prost et al., 2020; Sarkiss et al., 2018; Schoenfeld et al., 2016a, 2019, 2020a, 2020b; Switlyk et al., 2015; Uei and Tokuhashi, 2020; Zang et al., 2019). Study name, year published, level of evidence, design, sample size, patient makeup including sex and age, albumin cutoff used (with dichotomous or continuous levels), cohort incidence of hypoalbuminemia, treatment type, outcomes studied, and findings for each article are summarized in Table 1. Eight percent of studies (3 of 38) were considered level II evidence as defined by both retrospective and prospective cohort components (Yang et al., 2022; Prost et al., 2020; Zang et al., 2019), and 92% of studies (35 of 38) were considered level III evidence as defined by a retrospective cohort design (De et al., 2022; Boaro et al., 2020; Bongers et al., 2022; Cook and Baker, 2020; DiSilvestro et al., 2021; Dohzono et al., 2019; Ehresman et al., 2021; Gelfand et al., 2021; Ghori et al., 2015; Guo et al., 2003; Han et al., 2016; Hersh et al., 2018; Hussain et al., 2019; Karhade et al., 2016, 2019a, 2019b, 2022; Kato et al., 2016; Kumar et al., 2015; Liu et al., 2018; Lun et al., 2019; McPhee et al., 1998; Molho et al., 2022; Ogihara et al., 2006; Oh et al., 2012; Paulino et al., 2019; Pennington et al., 2021a, 2021b; Sarkiss et al., 2018; Schoenfeld et al., 2016a, 2019, 2020a, 2020b; Switlyk et al., 2015; Uei and Tokuhashi, 2020). A total of 21,401 patients across all studies were included, of which 6967 underwent both operative and nonoperative treatment (14 of 38 studies) (Yang et al., 2022; Cook and Baker, 2020; Dohzono et al., 2019; Ghori et al., 2015; Han et al., 2016; Karhade et al., 2022; Kato et al., 2016; Liu et al., 2018; McPhee et al., 1998; Schoenfeld et al., 2019, 2020a, 2020b; Uei and Tokuhashi, 2020; Zang et al., 2019), 13,883 underwent operative intervention only (n = 21) (De et al., 2022; Boaro et al., 2020; Bongers et al., 2022; DiSilvestro et al., 2021; Ehresman et al., 2021; Gelfand et al., 2021; Hersh et al., 2018; Hussain et al., 2019; Karhade et al., 2016, 2019a, 2019b; Kumar et al., 2015; Lun et al., 2019; Molho et al., 2022; Ogihara et al., 2006; Oh et al., 2012; Paulino et al., 2019; Pennington et al., 2021a, 2021b; Prost et al., 2020; Sarkiss et al., 2018), 491 received nonoperative management only (n = 2)^48,50^, and 60 received no treatment (n = 1)^25^. The following primary outcomes were evaluated in included studies: survival and/or mortality, complications, reoperation, readmission, length of hospital stay, ambulation, functional status, non-routine discharge, blood loss, and transfusion.

**Table 1:** Summary of 38 studies included in analysis.

Study	Level of Evidence	Design (Sample Size)	Demographic Makeup	Albumin Cutoff Used	Hypoalbuminemia Incidence	Treatment	Results
McPhee et al. (1998)	III	Retrospective cohort (53)	Male sex: 30 (57%) Mean age: 54 years	<3.5 g/dL	13 (25%)	Operative, nonoperative	Wound complication: Wound complication group had lower mean serum albumin (3.7 vs 4.0 g/dL; p = 0.032). Hypoalbuminemia not associated with wound complication on Chi-square (p = 0.135).
Guo et al. (2003)	III	Retrospective cohort (60)	Male: NR Age ≤60: 35 (58%)	≤3.5 g/dL	37 (62%)	None	Survival: Albumin level not associated with survival in univariate analysis.
Ogihara et al. (2006)	III	Retrospective cohort (114)	Male: 69 (61%) Mean age: 65 years	<3.0 g/dL	11 (10%)	Operative only	Survival: Low albumin associated with shorter mean NSCLC survival in multivariate analysis. Low albumin associated with shorter mean SCLC survival in univariate analysis.
Oh et al. (2012)	III	Retrospective cohort (52)	Male: 20 (38%) Mean age: 61 years	<3.5 g/dL	21 (40%)	Operative only	Survival: Low albumin associated with survival in multivariate analysis (HR 1.93; 95% CI 1.07–3.50; p = 0.028)
Ghori et al. (2015)	III	Retrospective cohort (307)	Male: 185 (58%) Mean age: 60 years	≥3.5 g/dL	128 (42%)	Operative, nonoperative	Survival: High albumin associated with 1-year survival in multivariate analysis (OR 2.80; 95% CI 1.66–4.72; p < 0.001)
Switlyk et al. (2015)	III	Retrospective cohort (173)	Male: 97 (56%) Median age: 65 years	<3.0 g/dL	13 (8%)	Nonoperative only	Survival: Low albumin associated with survival in multivariate analysis (HR 2.9; 95% CI 1.5–5.6; p = 0.001)
Kumar et al. (2015)	III	Retrospective cohort (98)	Male: 51 (52%) Mean age: 60 years	<3.5 g/dL	40 (42%)	Operative only	Wound complication: Albumin level not associated wound infection on multivariate analysis.
Schoenfeld et al., 2016a, Schoenfeld et al., 2016b	III	Retrospective cohort (318)	Male: 185 (58%) Mean age: 60 years	≥3.5 g/dL	128 (42%)	Nonoperative only	Survival: High albumin associated with 30-day survival in multivariate analysis (OR 9.0; 95% CI 3.1–26.6; p < 0.001). High albumin associated with 90-day survival in multivariate analysis (OR 3.9; 95% CI 2.2–6.8).
Han et al. (2016)	III	Retrospective cohort (37)	Male: 14 (38%) Mean age: 46 years	≤3.5 g/dL	6 (16%)	Operative, nonoperative	Survival: Albumin level not associated with survival on univariate analysis.
Kato et al. (2016)	III	Retrospective cohort (36)	Male: 26 (62%) Mean age: 59 years	<4.0 g/dL	20 (56%)	Operative, nonoperative	Survival: Albumin level not associated with survival on univariate analysis.
Karhade et al. (2016)	III	Retrospective cohort (2207)	Male: 1203 (55%) Age <46: 25% Age 46–58: 25% Age 58–67: 25% Age >67: 25%	<3.5 g/dL	355 (16%)	Operative only	Complications: Albumin level not associated with complications in multivariate analysis. Reoperation: Albumin level not associated with unplanned reoperation in multivariate analysis. Readmission: Albumin level not associated with unplanned readmission in multivariate analysis.
Hersh et al. (2018)	III	Retrospective cohort (616)	Male: 384 (62%) Age <51: 166 (27%) Age 51–60: 153 (25%) Age 61–70: 166 (27%) Age 71–80: 96 (16%) Age >80: 34 (6%)	<3.5 g/dL	189 (31%)	Operative only	Survival: Low albumin associated with 30-day mortality in multivariate analysis (OR 3.6; 95% CI 1.8–7.0; p = 0.000). Complications: Low albumin associated with 30-day complications in multivariate analysis (75% vs 39%; p < 0.001)
Sarkiss et al. (2018)	III	Retrospective cohort (300)	Male: 182 (61%) Age <51: 67 (22%) Age 51–60: 76 (25%) Age 61–70: 97 (32%) Age 71–80: 41 (14%) Age >80: 19 (6%)	<3.5 g/dL	51 (17%)	Operative only	Complications: Low albumin associated with 30-day complications on univariate analysis (39% vs 18%; p = 0.002).
Liu et al. (2018)	III	Retrospective cohort (54)	Male: 29 (54%) Age ≥60: 54 (100%)	≤3.5 g/dL	21 (39%)	Operative, nonoperative	Survival: Albumin level not associated with survival on univariate analysis. Functional Status: Albumin level not associated with functional outcome in univariate analysis.
Dohzono et al. (2019)	III	Retrospective cohort (78)	Male: 44 (56%) Mean age: 68 years	<3.7 g/dL	NR	Operative, nonoperative	Survival: Abnormal laboratory data, such as low albumin, associated with survival in multivariate analysis (HR 4.84; 95% CI 1.63–14.4; p = 0.005).
Schoenfeld et al. (2019)	III	Retrospective cohort (571)	Male: 272 (48%) Mean age: 58 years	≤3.5 g/dL	238 (42%)	Operative, nonoperative	Survival: Low albumin associated with 6-month mortality in multivariate analysis (OR 3.59; 95% CI 2.74–4.62). Low albumin associated with 1-year mortality in multivariate analysis (OR 3.21; 95% CI 2.38–4.33). Complications: Low albumin associated with complications in multivariate analysis (OR 1.37; 95% CI 1.04–1.75). Ambulation: Low albumin associated with 6-month non-ambulatory status in multivariate analysis (OR 4.85; 95% CI 2.74–8.10). Readmission: Low albumin associated with readmissions in multivariate analysis (OR 1.41; 95% CI 1.15–1.71).
Hussain et al. (2019)	III	Retrospective cohort (1498)	Male: 644 (43%) Age ≥65: 400 (41%)	<3.5 g/dL	512 (34%)	Operative only	Survival: Low albumin associated with 30-day mortality in multivariate analysis (OR 5.20; 95% CI 3.36–8.04; p < 0.001). Complication: Low albumin associated with 30-day complications in multivariate analysis (OR 3.16; 95% CI 2.44–4.10; p < 0.001). Sepsis: Low albumin associated with 30-day sepsis in multivariate analysis (OR 3.05; 95% CI 1.88–4.92; p < 0.001). Transfusion: Low albumin associated with 30-day intra- or postoperative transfusion in multivariate analysis (OR 1.43; 95% CI 1.11–1.83; p = 0.005). LOS: Low albumin associated with prolonged LOS in multivariate analysis (OR 4.32; 95% CI 3.31–5.63; p < 0.001). Discharge disposition: Low albumin associated with 30-day non-home discharge in multivariate analysis (OR 2.92; 95% CI 2.28–3.73; p < 0.001). Readmission: Low albumin associated with 30-day readmission in multivariate analysis (OR 0.18; 95% CI 0.05–0.63; p = 0.007).
Karhade et al., 2019b, Karhade et al., 2019a	III	Retrospective cohort (732)	Male: 426 (58%) Mean age: 61 years	≤3.8 g/dL	Median: 3.80 IQR: 3.4–4.2	Operative only	Survival: Low albumin was the most important variable for prediction of 90-day mortality. Low albumin was one of the most important variables for prediction of 1-year mortality.
Karhade et al., 2019b, Karhade et al., 2019a	III	Retrospective cohort (1790)	Male: 1093 (61%) Age <65: 1017 (57%) Age 65–79: 682 (38%) Age >80: 91 (5%)	<3.5 g/dL	553 (31%)	Operative only	Survival: Low albumin was more common in the 30-day mortality group (58% vs 28%). Low albumin was a significant predictor of 30-day mortality.
Lun et al. (2019)	III	Retrospective cohort (169)	Male: 102 (60%) Mean age: 60 years	<3.5 g/dL	17 (10%)	Operative only	Survival: Low albumin associated with survival in multivariate analysis (HR 6.9; 95% CI 2.5–19.1; p < 0.001).
Paulino et al. (2019)	III	Retrospective cohort (647)	Male: 375 (58%) Mean age: 60 years	Continuous	Median: 3.8 g/dL IQR: 3.4- – 4.2 g/dL	Operative only	Complications: Lower albumin level associated with 30-day complications in multivariate analysis (OR 0.69; 95% 0.49–0.96; p = 0.021).
Zang et al. (2019)	II	Retrospective and prospective cohort (239)	Male: 121 (51%) Age ≤60: 132 (55%) Age >60: 107 (45%)	>3.5 g/dL	100 (42%)	Operative, nonoperative	Survival: Albumin level not associated with survival on multivariate analysis.
Boaro et al. (2020)	III	Retrospective cohort (1176)	Male: 776 (66%) Mean age: 62 years	<3.5 g/dL	NR	Operative only	Complications: Albumin level not associated with major and minor complications on multivariate analysis.
Schoenfeld et al., 2020a, Schoenfeld et al., 2020b	III	Retrospective cohort (834)	Male: 384 (46%) Mean age: 57 years	≤3.5 g/dL	225 (27%)	Operative, nonoperative	Reoperation: Albumin level not associated with revision surgery in univariate analysis.
Schoenfeld et al., 2020a, Schoenfeld et al., 2020b	III	Retrospective cohort (1216)	Male: 608 (50%) Mean age: 58 years	>3.5 g/dL	303 (25%)	Operative, nonoperative	Survival: High albumin associated with survival (HR 0.54; 95% CI 0.45–0.64; p < 0.001) in multivariate analysis. High albumin associated with 6-month mortality (OR 0.3; 95% CI 0.2–0.4) and 1-year mortality (OR 0.4; 95% CI 0.3–0.5) on multivariate analysis. Treatment failure: High albumin associated with decreased treatment failure (OR 0.3; 95% CI 0.2–0.5) on multivariate analysis. Complications: Albumin level not associated with complications in multivariate analysis. Readmissions: Albumin level not associated with readmissions in multivariate analysis.
Uei and Tokuhashi (2020)	III	Retrospective cohort (62)	Male: 56 (90%) Age ≤69: 37 (60%) Age ≥70: 25 (40%)	≥3.5 g/dL	34 (55%)	Operative, nonoperative	Survival: High albumin associated with survival in multivariate analysis (HR 0.4; 95% CI 0.2–0.8; p = 0.010).
Prost et al. (2020)	II	Prospective cohort (264)	Male: 149 (56%) Mean age: 64 years	<3.5 g/dL	63 (29%)	Operative only	Survival: Low albumin associated with mortality in univariate analysis (OR 0.54; p < 0.001). Complications: Albumin level not associated with complications in univariate analysis.
Cook and Baker (2020)	III	Retrospective cohort (106)	Male: 66 (62%) Mean age: 65 years	>3.5 g/dL	NR	Operative, nonoperative	Survival: High albumin associated with survival in multivariate analysis (HR 0.5; 95% CI 0.3–0.7; p < 0.001).
De Meue et al. (2021)	III	Retrospective cohort (62)	Male: 41 (66%) Median age: 63 years	Continuous	Median: 4.3 g/dL IQR: 4.0–4.4 g/dL	Operative only	Survival: Higher albumin level associated with survival in multivariate analysis (HR 0.81; 95% CI 0.70–0.92; p = 0.002).
Ehresman et al. (2021)	III	Retrospective cohort (346)	Male: 186 (54%) Mean age: 57 years	Continuous	NR	Operative only	Discharge disposition: Lower albumin level associated with non-routine discharge in multivariate analysis (OR 0.4 g/dL; p < 0.001). LOS: Albumin level not associated with length of hospital stay in multivariate analysis.
DiSilvestro et al. (2021)	III	Retrospective cohort (2094)	Male: 1275 (61%) Mean age: 59 years	<3.5 g/dL	1156 (55%)	Operative only	Survival: Low albumin associated with 30-day mortality in multivariate analysis (OR 1.92; 95% CI 1.23–3.01; p = 0.004).
Gelfand et al. (2021)	III	Retrospective cohort (700)	Male: 451 (64%) Mean age: 61 years	≥3.5 g/dL (normal) 2.6–3.4 g/dL (mild) ≤2.5 g/dL (severe)	Normal: 448 (64%) Mild: 207 (30%) Severe: 45 (6%)	Operative only	Survival: Mild hypoalbuminemia associated with 30-day mortality in multivariate analysis (OR 1.7; 95% CI 1.0–3.0; p = 0.050). Severe hypoalbuminemia associated with 30-day mortality in multivariate analysis (OR 6.2; 95% CI 2.8–13.5; p < 0.001).
Pennington et al., 2021a, Pennington et al., 2021b	III	Retrospective cohort (350)	Male: 186 (53%) Mean age: 57 years	Continuous	Mean: 4.0 g/dL SD: 0.7 g/dL	Operative only	Complications: Albumin level associated with VTE in multivariate analysis (OR 0.4; 95% CI 0.2–0.8; p = 0.007). Albumin level associated with PE in multivariate analysis (OR 0.3; 95% CI 0.2–0.7; p = 0.003).
Pennington et al., 2021a, Pennington et al., 2021b	III	Retrospective cohort (274)	Male: 145 (53%) Mean age: 57 years	Continuous	NR	Operative only	Blood loss: Albumin level associated with intraoperative blood loss in multivariate analysis. (b −244.9 per g/dL, p = 0.011).
Karhade et al. (2022)	III	Retrospective cohort (3001)	Male: 1665 (55%) Mean age: 64 years	Continuous	Median: 3.7 g/dL IQR: 3.3–4.1 g/dL	Operative, nonoperative	Survival: Albumin level was the most important predictor of 6-week mortality.
Bongers et al. (2022)	III	Retrospective cohort (196)	Male: 123 (63%) Mean age: 62 years	Continuous	Median: 3.8 g/dL IQR: 3.4–4.2 g/dL	Operative only	Survival: Albumin level was associated with 90-day mortality in multivariate analysis (HR 0.20; 95% CI 0.11–0.38; p < 0.01). Albumin level was associated with 1-year mortality in multivariate analysis (HR 0.32; 95% CI 0.22–0.48; p < 0.01).
Molho et al. (2022)	III	Retrospective cohort (198)	Male: 121 (61%) Mean age: 65 years	≤3.5 g/dL	No complication group: 72 (47%) Complication group: 25 (61%)	Operative only	Complications: Low albumin associated with wound complications in multivariate4 analysis (OR 2.3; 95% CI 1.0–5.2; p = 0.044).
Yang et al. (2022)	II	Retrospective and prospective cohort (373)	Male: 248 (66%) Mean age: 55 years	Dichotomous (NR)	185 (50%)	Operative, nonoperative	Survival: Albumin level not associated with survival in multivariate analysis.

NR: not reported; NSCLC: non-small cell lung cancer; SCLC: small-cell lung cancer; HR: hazard ratio; CI: confidence interval; OR: odds ratio; IQR: interquartile range; SD: standard deviation; VTE: venous thromboembolism.

A total of 76% of studies (29 of 38) evaluated albumin level as a dichotomous variable (Yang et al., 2022; Boaro et al., 2020; Cook and Baker, 2020; DiSilvestro et al., 2021; Dohzono et al., 2019; Ghori et al., 2015; Guo et al., 2003; Han et al., 2016; Hersh et al., 2018; Hussain et al., 2019; Karhade et al., 2016, 2019a, 2019b; Kato et al., 2016; Kumar et al., 2015; Liu et al., 2018; Lun et al., 2019; McPhee et al., 1998; Molho et al., 2022; Ogihara et al., 2006; Oh et al., 2012; Prost et al., 2020; Sarkiss et al., 2018; Schoenfeld et al., 2016a, 2019, 2020a, 2020b; Switlyk et al., 2015; Uei and Tokuhashi, 2020; Zang et al., 2019), 18% (n = 7) evaluated albumin level as a continuous variable (De et al., 2022; Bongers et al., 2022; Ehresman et al., 2021; Karhade et al., 2022; Paulino et al., 2019; Pennington et al., 2021a, 2021b), and 3% (n = 1) sub-divided albumin levels into normal, mildly low, and severely low (Gelfand et al., 2021). One study utilized albumin as a dichotomous variable (normal vs. hypoalbuminemia) but did not define a cutoff value (Yang et al., 2022). Amongst studies that evaluated albumin as a dichotomous variable, 33% (4516 of 13.733) of patients across all studies were defined as having hypoalbuminemia based on respective study predefined cutoff values. Cutoff values defining hypoalbuminemia ranged from 3.0 g/dL to 4.0 g/dL. The distribution of studies defining hypoalbuminemia based on dichotomous values is as follows: 5% of studies used 3.0 g/dL (2 of 38), 66% of studies used 3.5 g/dL (n = 25), 3% of studies used 3.7 g/dL (n = 1), 3% of studies used 3.8 g/dL (n = 1), and 3% of studies used 4.0 g/dL (n = 1). One study that defined hypoalbuminemia at the <3.5 g/dL cutoff further defined mild hypoalbuminemia as 2.6–3.4 g/dL and severe hypoalbuminemia as ≤ 2.5 g/dL.

#### 3.1.1 Survival and mortality

A total of 71% of studies (27 of 38) evaluated the association between albumin and survival/mortality as a primary outcome in patients with spinal metastases, which was the most common outcome examined across all studies (Yang et al., 2022; De et al., 2022; Bongers et al., 2022; Cook and Baker, 2020; DiSilvestro et al., 2021; Dohzono et al., 2019; Gelfand et al., 2021; Ghori et al., 2015; Guo et al., 2003; Han et al., 2016; Hussain et al., 2019; Karhade et al., 2019a, 2019b, 2022; Kato et al., 2016; Lun et al., 2019; Ogihara et al., 2006; Oh et al., 2012; Prost et al., 2020; Schoenfeld et al., 2016a, 2020a; Switlyk et al., 2015; Uei and Tokuhashi, 2020; Zang et al., 2019). Of these, 74% of studies (20 of 27) found a significant association between albumin levels and survival/mortality.

Greater risk of short-term mortality was additionally found to be impacted by hypoalbuminemia in 48% of studies evaluating survival (13 of 27), including 30-day mortality in 22% (6 of 27), 6-week mortality in 4% (1 of 27), 90-day mortality in 11% (3 of 27), 6-month mortality in 7% (2 of 27), and 1-year mortality in 15% (4 of 27). Overall, hypoalbuminemia was generally found to be associated with shorter survival time and increased risk of mortality, while high albumin trended towards longer survival times and decreased risk of short-term mortality. Notably, all three studies utilizing a continuous albumin level to study survival found significant association between the two even after adjusting for potential confounding variables, with higher albumin level decreasing risk of mortality from a range of 0.2–0.81 (De et al., 2022; Bongers et al., 2022; Karhade et al., 2022). Twenty-four percent of studies (7 of 27) found no association between albumin level and survival/mortality.

#### 3.1.2 Complications

A total of 13 studies (34%; 13 of 38) included post-treatment complications as a studied primary outcome in analysis of spinal metastasis patient cohorts (Boaro et al., 2020; Hersh et al., 2018; Hussain et al., 2019; Karhade et al., 2016; Kumar et al., 2015; McPhee et al., 1998; Molho et al., 2022; Paulino et al., 2019; Pennington et al., 2021b; Prost et al., 2020; Sarkiss et al., 2018; Schoenfeld et al., 2019, 2020a). Of these studies, 54% (7 of 13) reported a significant association between albumin level and complications (Hersh et al., 2018; Hussain et al., 2019; Molho et al., 2022; Paulino et al., 2019; Pennington et al., 2021a; Sarkiss et al., 2018; Schoenfeld et al., 2019), while 46% of studies (6 of 13) demonstrated no relationship between albumin and complications (Boaro et al., 2020; Karhade et al., 2016; Kumar et al., 2015; McPhee et al., 1998; Prost et al., 2020; Schoenfeld et al., 2020a). An albumin cutoff level of 3.5 g/dL, used to define either high or low albumin in dichotomous analysis, was utilized in 85% of these studies (11 of 13) (Boaro et al., 2020; Hersh et al., 2018; Hussain et al., 2019; Karhade et al., 2016; Kumar et al., 2015; McPhee et al., 1998; Molho et al., 2022; Prost et al., 2020; Sarkiss et al., 2018; Schoenfeld et al., 2019, 2020a). A continuous analysis of albumin was used in only 15% of studies (2 of 13) evaluating complication outcomes, both of which were able to correlate albumin level with decreased risk of complications such as venous thromboembolism, pulmonary embolism, infection, and others (Paulino et al., 2019; Pennington et al., 2021b).

One study assessed sepsis as a specific 30-day postoperative outcome, finding low albumin <3.5 g/dL to be associated with increased risk of sepsis in multivariate analysis (Hussain et al., 2019). One study further investigated the effect of albumin level on treatment failure (defined as survival <6 months or decline in ambulatory function), which demonstrated high albumin >3.5 g/dL to decrease risk of treatment failure after adjusting for confounding variables (Schoenfeld et al., 2020a).

#### 3.1.3 Readmission and reoperation

The potential association between albumin level and unplanned hospital readmission was assessed in 4 studies (11%; 4 of 38) (Hussain et al., 2019; Karhade et al., 2016; Schoenfeld et al., 2019, 2020a). Fifty percent of these studies (2 of 4) reported no significant association between low preoperative albumin and unplanned readmission after adjusting for potential confounders (Karhade et al., 2016; Schoenfeld et al., 2020a). Of the two other studies that found a significant association between albumin level and readmission, one reported low albumin to increase risk of readmission while the other reported low albumin to decrease risk of 30-day readmission in adjusted analyses (Hussain et al., 2019; Schoenfeld et al., 2019). All four studies analyzing readmission used an albumin level cutoff of 3.5 g/dL.

Two studies additionally evaluated reoperation as a primary outcome in spinal metastasis patients using an albumin cutoff value of <3.5 g/dL, both of which found no association between hypoalbuminemia and unplanned revision surgery (Karhade et al., 2016; Schoenfeld et al., 2020b).

#### 3.1.4 Length of hospital stay

Length of hospital stay was evaluated in two studies, one of which utilized a continuous albumin level and while the other utilized discrete hypoalbuminemia as defined by albumin <3.5 g/dL (Ehresman et al., 2021; Hussain et al., 2019). Although hypoalbuminemia <3.5 g/dL was found to be associated with prolonged length of stay in adjusted analysis (Hussain et al., 2019), albumin level was not found to have any association with length of hospital stay when evaluated as in a continuous fashion (Ehresman et al., 2021).

#### 3.1.5 Ambulation and functional status

One study included functional status as a primary outcome and one study included ambulation as a primary outcome in relation to albumin level (Liu et al., 2018; Schoenfeld et al., 2019). While both studies utilized a hypoalbuminemia cutoff of albumin 3.5 g/dL, one study found low albumin to be associated with increased 6-month risk of non-ambulation in multivariate analysis (Schoenfeld et al., 2019), while the other reported no association between albumin level and functional outcome (Liu et al., 2018).

#### 3.1.6 Discharge disposition

Discharge disposition was included as a primary outcome in two studies, both of which found a statistically significant association between albumin level and non-routine discharge (Ehresman et al., 2021; Hussain et al., 2019). Hypoalbuminemia <3.5 g/dL was found to increase odds of 30-day non-home discharge in multivariate analysis (Hussain et al., 2019), and lower albumin level on a continuous scale was similarly found to be associated with greater odds of non-routine discharge in adjusted results (Ehresman et al., 2021).

#### 3.1.7 Blood loss

Only one study evaluated the association between albumin level and blood loss (Pennington et al., 2021a), while another study evaluated the association between albumin level and transfusion (Hussain et al., 2019). Both studies described a statistically significant relationship between albumin and these bleeding-related complications: albumin level as a continuous variable was associated with greater intraoperative blood loss, and hypoalbuminemia <3.5 g/dL was associated with increased odds of 30-day intraoperative or postoperative transfusion in adjusted analyses.

## 4 Discussion

### 4.1 Key findings

Hypoalbuminemia is associated with inflammation and malnutrition, both of which are common in cancer patients. Furthermore, low albumin levels been associated with poor outcomes in almost every primary cancer type, including colorectal, cervical, hepatocellular, gastric, lung, ovarian, and nasopharyngeal cancers (Kengsakul et al., 2022; Christina et al., 2023; Yoshikawa et al., 2022; Crumley et al., 2010; Stares et al., 2021; Yang et al., 2020). In this scoping review, we aimed to identify the most used cutoff value to define hypoalbuminemia in patients with spinal metastases, and explore the association between serum albumin level and outcomes after operative and non-operative management.

The findings of this study suggest that a threshold of 3.5 g/dL seems most appropriate to define hypoalbuminemia in patients with spinal metastases. However, studies that evaluated albumin as a continuous variable consistently demonstrated a level-dependent effect, with lower albumin levels associated with worse outcomes for both patients undergoing operative and non-operative management. The most consistent significant association explored in the literature was between low albumin and survival, with 74% of studies exploring this association reporting a significant relationship between hypoalbuminemia and either shorter survival or increased mortality. This association was observed at both: fixed end-points (such as 30-day, 90-day, 1-year mortality) as well as upon exploring overall survival. In contrast, the evidence supporting an association between hypoalbuminemia and other clinical outcomes was more inconsistent. More than half of the studies evaluating postoperative complications found a significant relationship with low albumin levels, while the findings were inconsistent for other explored outcomes such as readmission, reoperation, length of hospital stay, and ambulation. Only a small number of studies assessed the impact of albumin on discharge disposition, blood loss and related transfusion requirement, with these studies reporting a significant association.

**Fig. 2:**
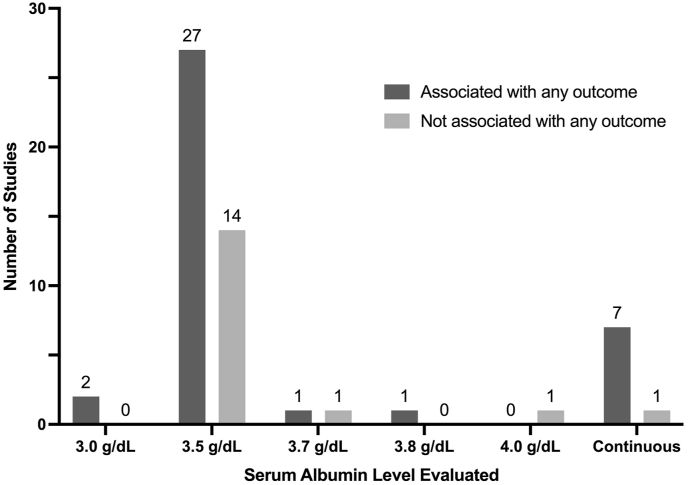
Distribution of studies utilizing different albumin measures categorized by association with any outcome.

**Fig. 3:**
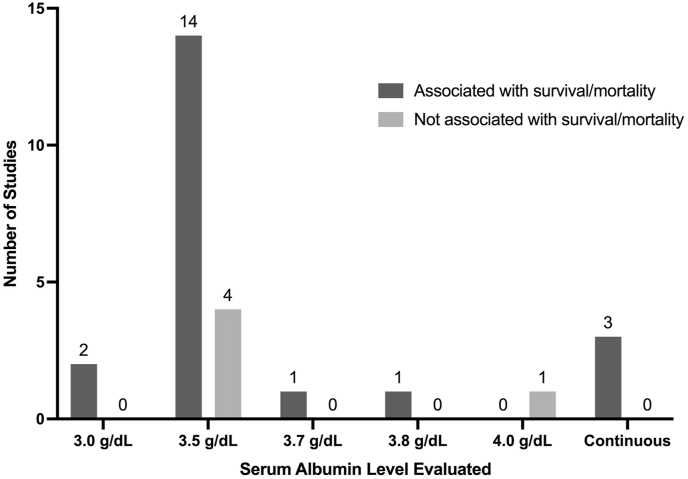
Distribution of studies utilizing different albumin measures categorized by association with survival/mortality.

**Fig. 4:**
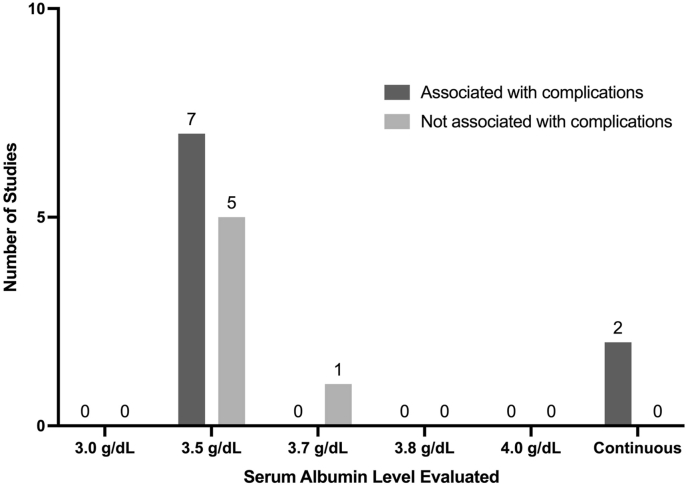
Distribution of studies utilizing different albumin measures categorized by association with complications.

The association between hypoalbuminemia and outcomes in patients with spinal metastases has been a key area of interest in the cancer literature (Perrin and Laxton, 2004). Out of the studies included in this review, the most commonly evaluated outcome was survival and/or mortality, followed by complications, reoperation, readmission, length of hospital stay, ambulation, functional status, discharge disposition, and lastly blood loss. Despite most studies finding hypoalbuminemia to be associated with at least one of the examined outcomes, the serum albumin cutoff level used to define hypoalbuminemia in our included studies was inconsistent. The proportion of studies utilizing each unique hypoalbuminemia cutoff level is summarized in Fig. 2, stratified by studies which found some association vs. no association with primary outcomes. While no discrete pattern can be established from these studies, a serum albumin threshold of 3.5 g/dL is utilized by many groups and institutions to define low albumin in a clinical setting. In those that utilized this standard cutoff value, approximately twice as many studies reported an independent and significant association between hypoalbuminemia and any outcome when compared to studies that found no association with outcomes (27 vs 14 studies). This trend was persistent amongst the two most studied outcomes, survival/mortality [Fig. 3] and complications [Fig. 4], where significantly more studies found a positive association between hypoalbuminemia and outcomes when compared to those that did not find any association between the two. Out of the two studies that utilized a lower serum albumin cutoff than standard (3.0 g/dL), both reported a significant association with their studied outcome (Ogihara et al., 2006; Switlyk et al., 2015). However, as the reported serum albumin cutoff value increased to 3.7 g/dL and greater, inconsistent findings became more apparent, with 2 studies finding significant association with a poor outcome and 2 studies finding no association with outcomes (Dohzono et al., 2019; Karhade et al., 2019a; Kato et al., 2016). Finally, the utilization of serum albumin as a continuous variable displayed the most consistent results, of which all 3 studies evaluating survival and all 2 studies evaluating complications finding a significant association (De et al., 2022; Bongers et al., 2022; Ehresman et al., 2021; Karhade et al., 2022; Paulino et al., 2019; Pennington et al., 2021a, 2021b). From a clinical standpoint, dichotomizing patients into having or not having hypoalbuminemia is a more practical approach, and the current best available evidence suggests a threshold of 3.5 g/dL. However, it is imperative to also know that there seems to be a level-dependent effect, with lower albumin levels associated with worse outcome.

While it is possible that regional variance or differences in laboratory techniques define hypoalbuminemia at unique values, most of the studies with non-standard hypoalbuminemia cutoff values did not provide rationale for choosing their specified cutoff levels. As we learn more about the role that hypoalbuminemia plays in pathologic disease states and in cancer-related outcomes, it becomes crucial to establish consistent definitions so that future studies may draw reliable conclusions and recommend evidence-based clinical-decision making tools.

#### 4.1.1 Interpretation and generalizability

Albumin, the most abundant circulating protein in the human body, is produced in the liver and responds to physiologic stressors and/or signals including corticosteroids, insulin, and intravascular osmotic pressures to increase synthesis or catabolism (Rothschild et al., 1988; Nicholson et al., 2000). In addition to regulating plasma buffer homeostasis and transporting fat-soluble substances, albumin is responsible for scavenging of oxygen free radicals that are produced from inflammatory activity (Simpkins et al., 2004; Bourdon et al., 1999; Fanali et al., 2012). Hypoalbuminemia, as a result, can be observed as a symptom of underlying disease states associated with chronic inflammation, which include critical illnesses, infections, major surgery, and advanced cancer (Gatta et al., 2012). However, serum albumin has also been shown to respond to overall nutritional status, where reduced protein and caloric intake may decrease intrinsic hepatic albumin synthesis or reduce circulating albumin half-life through increased degradation (Soeters et al., 2019; Don and Kaysen, 2004). Cancer in particular has been shown to be associated with hypoalbuminemia through multiple pathologic mechanisms, including those associated with tumor-related inflammation and poor oral nutritional intake, where the true mechanism is likely to be a combination of the two (McMillan, 2009; Gradel, 2023; Gupta and Lis, 2010).

The pathophysiology between advanced metastatic disease and hypoalbuminemia is complex and has not yet been completely defined. Current understanding of low serum albumin in cancer is the result of a combination of increased vascular permeability, increased degradation/decreased albumin half-life, and a decreased distribution volume throughout the body (Soeters et al., 2019). Inflammatory cytokines, including VEGF, IL-1, IL-6, and TNF-a, are produced by tumor cells that promote cellular growth and differentiation, but concurrently cause endothelial cell dysfunction leading to greater capillary hyperpermeability (Soeters et al., 2019; Sawant et al., 2013; Le et al., 2011; Hirano, 2021). Albumin thus escapes the intravascular compartment and triggers increased hepatic synthesis to replace the lost intravascular albumin volume (Fearon et al., 1998; Barle et al., 2006). Oxygen free radical production by the tumor microenvironment additionally causes increased albumin oxidation/glycation in peripheral spaces, thus leading to reduced antioxidant capacity and greater albumin breakdown either through hepatic endocytosis or direct tumor cell absorption (Gatta et al., 2012; Bourdon et al., 1999; Kamphorst et al., 2015). From a nutritional standpoint, inflammatory cytokines IL-1, IL-6, and TNF-a have been found to mimic leptin signaling, diminishing expected compensatory hunger responses and leading to decreased overall oral intake (Suzuki et al., 2013). However, prior studies have not established a clear association between nutritional supplementation and serum albumin response, even when administered in critically ill patients such as those with advanced cancer (Gradel, 2023). Furthermore, normal serum albumin levels have been repeatedly established in patients suffering from poor nutritional status and/or anorexia, which leads us to consider inflammation as a primary mechanism to hypoalbuminemia in oncologic disease (Soeters et al., 2019; McMillan et al., 2001; Lee et al., 2015).

In compiling a comprehensive summary of literature evaluating the prognostic utility of hypoalbuminemia in spinal metastasis patients, our aim was to provide greater insight into the current utilization of serum albumin in this population. The ultimate impact of this work is broad but is likely to be specific to spinal tumor surgeons and oncology experts who may rely on a combination of patient-specific characteristics and biomarkers to plan medical optimization strategies and treatment courses. Incorporation of serum albumin and/or hypoalbuminemia into clinical outcome predictive models is not a novel phenomenon, as several of these prognostic indices already rely on patient albumin status to estimate survival and other measures of morbidity (Gradel, 2023). The Prognostic Nutritional Index (PNI), Gustave Roussy Immune (GRIM), and Glasgow Prognostic scores are a few such models that have been established and validated in patients with various malignancies; however, only one modern albumin-based scoring system is specific to patients with spinal metastatic disease (De la Garza Ramos et al., 2023; Li et al., 2020; Wu et al., 2023). The New England Spinal Metastasis Score (NESMS), incorporating a sum of weighted point values based on the presence of visceral metastases, primary tumor type, number of skeletal lesions, ambulatory status, and serum albumin level <3.5 g/dL was found to be predictive of short- and long-term survival as well as postoperative complications and length of hospital stay using large-scale patient databases (Schoenfeld et al., 2016b). As targeted immunotherapies, stereotactic radiation techniques, and surgical approaches continue to evolve in the oncologic space, personalized treatment regimens based on patient-specific risk factors will become fundamental to the clinical decision-making and informed consent processes (Sullivan et al., 2022). Balancing the risks of invasive treatment options with the benefits of reduced tumor burden and pain control will likely come to rely on prognostication tools that are specific to unique patient populations, such as those with spinal metastases. Identification of those with hypoalbuminemia, or poor nutritional status/inflammatory responses may trigger clinicians to identify underlying disease causes and optimize the pre- and post-treatment course to aid in recovery. However, it is important to consider serum albumin within the context of other established prognostic factors to provide reliable morbidity and mortality estimates.

#### 4.1.2 Treating hypoalbuminemia

Despite the strong apparent link between hypoalbuminemia and poor outcomes in a range of oncologic patients, the order of causality between acute illness and serum albumin level is still unclear (Garcia de Herreros et al., 2024). Exogenous human albumin supplementation in the perioperative period has been utilized to correct serum albumin abnormalities in certain cases, with some evidence of mortality and complication benefit in patients undergoing operations for head and neck disease as well as hip arthroplasty (Xu et al., 2021; Liu et al., 2015). However, the results of several randomized controlled trials failed to find clinically significant survival benefit following exogenous albumin supplementation in hypoalbuminemic patients, suggesting that treatment of potential underlying causes of inflammation or malnutrition is preferred to correcting hypoalbuminemia in inpatient settings (Wilkes and Navickis, 2001; Vincent et al., 2003; Foley et al., 1990). In the oncologic population, multidisciplinary approaches including aggressive nutritional supplementation and physical therapy regimens have been recommended to increase lean body mass and muscle function in efforts to reverse cancer-related cachexia, which aims to target underlying causes of hypoalbuminemia (Kose et al., 2021). Parenteral supplementation with glutamine has similarly shown positive effects on low serum albumin levels in surgical oncology patients, specifically in those with gastrointestinal cancers; however, these effects were inconsistently correlated postoperative outcomes in this population (Wu et al., 2021). Finally, nutritional supplementation has been found to improve and stabilize serum albumin levels in postoperative breast cancer patients undergoing simultaneous chemotherapeutic regimens, which may suggest that a multifaceted approach targeting both inflammatory and malnutrition states may aid in improving hypoalbuminemia and subsequent postoperative outcomes (Grupinska et al., 2021). Limited work currently exists in the literature regarding the role of albumin supplementation in the context of operative spinal metastatic disease and concurrent systemic chemotherapeutic treatments, however our review may aid in providing a foundation to develop albumin monitoring and supplementation protocols in this largely frail patient population.

#### 4.1.3 Future research direction

Given the findings of this scoping review, there are many critical avenues for future research to further elucidate the role of hypoalbuminemia as a prognostic factor in patients with spinal metastases. There is a need for well-designed prospective studies to more definitively establish the causal relationship between low serum albumin levels and adverse clinical outcomes in this population. Such studies should aim to disentangle the complex interplay between hypoalbuminemia, inflammation, nutritional status, and other potential confounding variables that may influence patient prognosis. Randomized controlled trials evaluating the impact of preoperative albumin supplementation on postoperative outcomes would be particularly valuable, as they could provide insight into whether correcting hypoalbuminemia can improve patient prognosis.

Additionally, future research should also explore the incorporation of serum albumin into novel decision-support tools and predictive models to assist clinicians in the complex decision-making process regarding surgical versus non-operative management of spinal metastases. By better understanding how albumin levels impact survival, functional outcomes, and quality of life, these prognostic models could empower shared decision-making involving clinicians and patients to optimize care and align treatment approaches with individual goals and preferences.

#### 4.1.4 Limitations

There were some inherent limitations to this review. First, while a total of 38 studies were included, the quality of studies was limited to LOE II and LOE III, with most articles utilizing a retrospective cohort design. Only three studies incorporated a prospective component, which underscores the importance of conducting further prospective work on this topic to establish reliable associations between albumin and outcomes in spinal oncology. Furthermore, the outcomes analyzed in these studies were variable and included a range of time endpoints. For this reason, a meta-analysis was not able to be performed, and limited our current work to a strictly narrative review. The strength of this review lies in our utilization of the PRISMA guidelines to ensure article accuracy and quality; however, reviews such as these are ultimately limited to the available literature. The inclusion of studies not limited in time, region, and language aided in expanding the scope of this topic so that an in-depth analysis of the effect of hypoalbuminemia on spinal oncology outcomes was able to be conducted.

## 5 Conclusions

Serum albumin level as a biomarker for inflammation and malnutrition has been extensively studied in cancer patients, with recent focus into specific oncologic populations such as those with spinal metastases. In this scoping review, we found that a threshold of 3.5 g/dL seems most appropriate to define hypoalbuminemia in this patient population. From a biologic standpoint, however, there is also evidence supporting a level-dependent effect, with lower albumin levels associated with shorter survival. Most reviewed studies found a significant association between serum albumin level and survival/mortality for both operative and non-operative management. Nevertheless, the association between serum albumin and other outcomes including complications was less consistent. Future prospective research examining the incorporation of albumin levels into the clinical decision-making process of patients with spinal metastases as well as the potential for perioperative optimization is needed.

## Previous presentations

None.

## Disclosures

None.

## IRB approval

Not needed.

## Patient consent

Not needed.

## Author contributions

Jessica Ryvlin and Rafael De la Garza Ramos contributed to the conception of the study idea; Jessica Ryvlin, Namal Seneviratne, Ali Haider Bangash, C. Rory Goodwin, Michael Weber, and Raphaële Charest-Morin contributed to the design of the study, acquisition of data, analysis and interpretation of data, and drafting the article; John H Shin, Anne L Versteeg, Mitchel S Fourman, Saikiran G Murthy, Yaroslav Gelfand, Reza Yassari, and Rafael De la Garza Ramos contributed to drafting the article, revising it critically for important intellectual content, and final approval of the version to be submitted.

## Funding

None.

## Declaration of competing interest

The authors declare that they have no known competing financial interests or personal relationships that could have appeared to influence the work reported in this paper.

## References

[bib1] Bakhsheshian J., Shahrestani S., Buser Z. (2022). The performance of frailty in predictive modeling of short-term outcomes in the surgical management of metastatic tumors to the spine. Spine J..

[bib2] Barle H., Hammarqvist F., Westman B. (2006). Synthesis rates of total liver protein and albumin are both increased in patients with an acute inflammatory response. Clin. Sci..

[bib3] Boaro A., Wells M., Chi J. (2020). A national surgical quality improvement program analysis of postoperative major and minor complications in patients with spinal metastatic disease. World Neurosurg.

[bib4] Bongers M.E.R., Groot O.Q., Buckless C.G. (2022). Body composition predictors of mortality on computed tomography in patients with spinal metastases undergoing surgical treatment. Spine J..

[bib5] Bourdon E., Loreau N., Blache D. (1999). Glucose and free radicals impair the antioxidant properties of serum albumin. FASEB J..

[bib6] Christina N.M., Tjahyanto T., Lie J.G. (2023). Hypoalbuminemia and colorectal cancer patients: any correlation?: a systematic review and meta-analysis. Medicine (Baltim.).

[bib7] Cook W.H., Baker J.F. (2020). Retrospective evaluation of prognostic factors in metastatic spine disease: serum albumin and primary tumour type are key. ANZ J. Surg..

[bib8] Crumley A.B., Stuart R.C., McKernan M., McMillan D.C. (2010). Is hypoalbuminemia an independent prognostic factor in patients with gastric cancer?. World J. Surg..

[bib9] De Meue E., Smeijers S., Langmans C., Clement P.M., Depreitere B. (2022). Identifying new predictive factors for survival after surgery for spinal metastases: an exploratory in-depth retrospective analysis. Acta Clin. Belg..

[bib10] De la Garza Ramos R., Ryvlin J., Hamad M.K. (2023). The prognostic nutritional index (PNI) is independently associated with 90-day and 12-month mortality after metastatic spinal tumor surgery. Eur. Spine J..

[bib11] DiSilvestro K.J., Veeramani A., McDonald C.L. (2021). Predicting postoperative mortality after metastatic intraspinal neoplasm excision: development of a machine-learning approach. World Neurosurg..

[bib12] Dohzono S., Sasaoka R., Takamatsu K., Hoshino M., Nakamura H. (2019). Prognostic value of paravertebral muscle density in patients with spinal metastases from gastrointestinal cancer. Support. Care Cancer.

[bib13] Don B.R., Kaysen G. (2004). Serum albumin: relationship to inflammation and nutrition. Semin. Dial..

[bib14] Ehresman J., Pennington Z., Feghali J. (2021). Predicting nonroutine discharge in patients undergoing surgery for vertebral column tumors. J. Neurosurg. Spine.

[bib15] Fanali G., di Masi A., Trezza V., Marino M., Fasano M., Ascenzi P. (2012). Human serum albumin: from bench to bedside. Mol. Aspect. Med..

[bib16] Fang L., Yan F.H., Liu C. (2021). Systemic inflammatory biomarkers, especially fibrinogen to albumin ratio, predict prognosis in patients with pancreatic cancer. Cancer Res Treat..

[bib17] Fearon K.C., Falconer J.S., Slater C., McMillan D.C., Ross J.A., Preston T. (1998). Albumin synthesis rates are not decreased in hypoalbuminemic cachectic cancer patients with an ongoing acute-phase protein response. Ann. Surg..

[bib18] Foley E.F., Borlase B.C., Dzik W.H., Bistrian B.R., Benotti P.N. (1990). Albumin supplementation in the critically ill. A prospective, randomized trial. Arch. Surg..

[bib19] Garcia de Herreros M., Laguna J.C., Padrosa J. (2024). Characterisation and outcomes of patients with solid organ malignancies admitted to the intensive care unit: mortality and impact on functional status and oncological treatment. Diagnostics.

[bib20] Gatta A., Verardo A., Bolognesi M. (2012). Hypoalbuminemia. Intern. Emerg. Med..

[bib21] Gelfand Y., De la Garza Ramos R., Nakhla J.P., Echt M., Yanamadala V., Yassari R. (2021). Predictive value of hypoalbuminemia and severe hypoalbuminemia in oncologic spine surgery. Clin. Neurol. Neurosurg..

[bib22] Ghori A.K., Leonard D.A., Schoenfeld A.J. (2015). Modeling 1-year survival after surgery on the metastatic spine. Spine J..

[bib23] Gradel K.O. (2023). Interpretations of the role of plasma albumin in prognostic indices: a literature review. J. Clin. Med..

[bib24] Grupinska J., Budzyn M., Mackowiak K. (2021). Beneficial effects of oral nutritional supplements on body composition and biochemical parameters in women with breast cancer undergoing postoperative chemotherapy: a propensity score matching analysis. Nutrients.

[bib25] Guo Y., Young B., Palmer J.L., Mun Y., Bruera E. (2003). Prognostic factors for survival in metastatic spinal cord compression: a retrospective study in a rehabilitation setting. Am. J. Phys. Med. Rehabil..

[bib26] Gupta D., Lis C.G. (2010). Pretreatment serum albumin as a predictor of cancer survival: a systematic review of the epidemiological literature. Nutr. J..

[bib27] Han S., Yang X., Jiang D. (2016). Surgical outcomes and prognostic factors in patients with diffuse large B-cell lymphoma-associated metastatic spinal cord compression. Spine.

[bib28] Hersh E.H., Sarkiss C.A., Ladner T.R. (2018). Perioperative risk factors for thirty-day morbidity and mortality in the resection of extradural thoracic spine tumors. World Neurosurg..

[bib29] Hersh A.M., Pennington Z., Hung B. (2022). Comparison of frailty metrics and the Charlson Comorbidity Index for predicting adverse outcomes in patients undergoing surgery for spine metastases. J. Neurosurg. Spine.

[bib30] Hirano T. (2021). IL-6 in inflammation, autoimmunity and cancer. Int. Immunol..

[bib31] Hussain A.K., Cheung Z.B., Vig K.S. (2019). Hypoalbuminemia as an independent risk factor for perioperative complications following surgical decompression of spinal metastases. Glob. Spine J..

[bib32] Kamphorst J.J., Nofal M., Commisso C. (2015). Human pancreatic cancer tumors are nutrient poor and tumor cells actively scavenge extracellular protein. Cancer Res..

[bib33] Karhade A.V., Vasudeva V.S., Dasenbrock H.H. (2016). Thirty-day readmission and reoperation after surgery for spinal tumors: a National Surgical Quality Improvement Program analysis. Neurosurg. Focus.

[bib34] Karhade A.V., Thio Q., Ogink P.T. (2019). Predicting 90-day and 1-year mortality in spinal metastatic disease: development and internal validation. Neurosurgery.

[bib35] Karhade A.V., Thio Q., Ogink P.T. (2019). Development of machine learning algorithms for prediction of 30-day mortality after surgery for spinal metastasis. Neurosurgery.

[bib36] Karhade A.V., Fenn B., Groot O.Q. (2022). Development and external validation of predictive algorithms for 6-week mortality in spinal metastasis using 4,304 patients from 5 institutions. Spine J..

[bib37] Kato S., Murakami H., Demura S. (2016). Spinal metastasectomy of renal cell carcinoma: a 16-year single center experience with a minimum 3-year follow-up. J. Surg. Oncol..

[bib38] Kengsakul M., Nieuwenhuyzen-de Boer G.M., Udomkarnjananun S., Kerr S.J., Niehot C.D., van Beekhuizen H.J. (2022). Factors predicting postoperative morbidity after cytoreductive surgery for ovarian cancer: a systematic review and meta-analysis. J. Gynecol. Oncol..

[bib39] Kim S., McClave S.A., Martindale R.G., Miller K.R., Hurt R.T. (2017). Hypoalbuminemia and clinical outcomes: what is the mechanism behind the relationship?. Am. Surg..

[bib40] Kose E., Wakabayashi H., Yasuno N. (2021). Polypharmacy and malnutrition management of elderly perioperative patients with cancer: a systematic review. Nutrients.

[bib41] Kumar S., Van Popta D., Rodrigues-Pinto R. (2015). Risk factors for wound infection in surgery for spinal metastasis. Eur. Spine J..

[bib42] Le Guelte A., Dwyer J., Gavard J. (2011). Jumping the barrier: VE-cadherin, VEGF and other angiogenic modifiers in cancer. Biol. Cell..

[bib43] Lee J.L., Oh E.S., Lee R.W., Finucane T.E. (2015). Serum albumin and Prealbumin in calorically restricted, nondiseased individuals: a systematic review. Am. J. Med..

[bib44] Li S.J., Zhao L., Wang H.Y. (2020). Gustave Roussy Immune Score based on a three-category risk assessment scale serves as a novel and effective prognostic indicator for surgically resectable early-stage non-small-cell lung cancer: a propensity score matching retrospective cohort study. Int. J. Surg..

[bib45] Liao C.K., Yu Y.L., Lin Y.C. (2021). Prognostic value of the C-reactive protein to albumin ratio in colorectal cancer: an updated systematic review and meta-analysis. World J. Surg. Oncol..

[bib46] Liberati A., Altman D.G., Tetzlaff J. (2009). The PRISMA statement for reporting systematic reviews and meta-analyses of studies that evaluate healthcare interventions: explanation and elaboration. BMJ.

[bib47] Liu M., Yang J., Yu X. (2015). The role of perioperative oral nutritional supplementation in elderly patients after hip surgery. Clin. Interv. Aging.

[bib48] Liu Y., Zhou R., Qin H., Liu S., Wang L. (2018). Prognostic factors and surgical outcome after decompressive surgery in aged patients with metastatic spinal cord compression. Int. J. Clin. Exp. Med..

[bib49] Luksanapruksa P., Buchowski J.M., Zebala L.P., Kepler C.K., Singhatanadgige W., Bumpass D.B. (2017). Perioperative complications of spinal metastases surgery. Clin. Spine Surg..

[bib50] Luksanapruksa P., Buchowski J.M., Hotchkiss W., Tongsai S., Wilartratsami S., Chotivichit A. (2017). Prognostic factors in patients with spinal metastasis: a systematic review and meta-analysis. Spine J..

[bib51] Lun D.X., Xu L.N., Wang F. (2019). Prognostic differences in patients with solitary and multiple spinal metastases. Orthop. Surg..

[bib52] McMillan D.C. (2009). Systemic inflammation, nutritional status and survival in patients with cancer. Curr. Opin. Clin. Nutr. Metab. Care.

[bib53] McMillan D.C., Watson W.S., O’Gorman P., Preston T., Scott H.R., McArdle C.S. (2001). Albumin concentrations are primarily determined by the body cell mass and the systemic inflammatory response in cancer patients with weight loss. Nutr. Cancer.

[bib54] McPhee I.B., Williams R.P., Swanson C.E. (1998). Factors influencing wound healing after surgery for metastatic disease of the spine. Spine.

[bib55] Molho N., Pereira-Duarte M., Estefan M. (2022). Wound-related complications in the surgical treatment of vertebral metastatic disease - a case series analysis. Rev. Española Cirugía Ortopédica Traumatol..

[bib56] Nicholson J.P., Wolmarans M.R., Park G.R. (2000). The role of albumin in critical illness. Br. J. Anaesth..

[bib57] Ogihara S., Seichi A., Hozumi T. (2006). Prognostic factors for patients with spinal metastases from lung cancer. Spine.

[bib58] Oh I.S., Kim S.I., Ha K.Y. (2012). Significant predictive values for the life expectancy in patients with spinal metastasis following surgical treatment. Eur. J. Orthop. Surg. Traumatol..

[bib59] Paulino Pereira N.R., Janssen S.J., van Dijk E. (2016). Development of a prognostic survival algorithm for patients with metastatic spine disease. J. Bone Joint Surg. Am..

[bib60] Paulino Pereira N.R., Ogink P.T., Groot O.Q. (2019). Complications and reoperations after surgery for 647 patients with spine metastatic disease. Spine J..

[bib61] Pennington Z., Ehresman J., Feghali J. (2021). A clinical calculator for predicting intraoperative blood loss and transfusion risk in spine tumor patients. Spine J..

[bib62] Pennington Z., Ehresman J., Schilling A. (2021). Influence of tranexamic acid use on venous thromboembolism risk in patients undergoing surgery for spine tumors. J. Neurosurg. Spine.

[bib63] Perrin R.G., Laxton A.W. (2004). Metastatic spine disease: epidemiology, pathophysiology, and evaluation of patients. Neurosurg. Clin..

[bib64] Prost S., Bouthors C., Fuentes S. (2020). Influence of preoperative biological parameters on postoperative complications and survival in spinal bone metastasis. A multicenter prospective study. J. Orthop. Traumatol.: Surg. Res..

[bib65] Rothschild M.A., Oratz M., Schreiber S.S. (1988). Serum albumin. Hepatology.

[bib66] Sarkiss C.A., Hersh E.H., Ladner T.R. (2018). Risk factors for thirty-day morbidity and mortality in extradural lumbar spine tumor resection. World Neurosurg..

[bib67] Sawant D.A., Tharakan B., Wilson R.L., Stagg H.W., Hunter F.A., Childs E.W. (2013). Regulation of tumor necrosis factor-alpha-induced microvascular endothelial cell hyperpermeability by recombinant B-cell lymphoma-extra large. J. Surg. Res..

[bib68] Schoenfeld A.J., Leonard D.A., Saadat E., Bono C.M., Harris M.B., Ferrone M.L. (2016). Predictors of 30- and 90-day survival following surgical intervention for spinal metastases: a prognostic study conducted at four academic centers. Spine.

[bib69] Schoenfeld A.J., Le H.V., Marjoua Y. (2016). Assessing the utility of a clinical prediction score regarding 30-day morbidity and mortality following metastatic spinal surgery: the New England Spinal Metastasis Score (NESMS). Spine J..

[bib70] Schoenfeld A.J., Ferrone M.L., Schwab J.H. (2019). Prognosticating outcomes and survival for patients with lumbar spinal metastases: results of a bayesian regression analysis. Clin. Neurol. Neurosurg..

[bib71] Schoenfeld A.J., Ferrone M.L., Passias P.G. (2020). Laboratory markers as useful prognostic measures for survival in patients with spinal metastases. Spine J..

[bib72] Schoenfeld A.J., Schwab J.H., Ferrone M.L. (2020). Non-operative management of spinal metastases: a prognostic model for failure. Clin. Neurol. Neurosurg..

[bib73] Simpkins C.O., Little D., Brenner A., Hill J.A., Griswold J.A. (2004). Heterogeneity in the effect of albumin and other resuscitation fluids on intracellular oxygen free radical production. J. Trauma.

[bib74] Soeters P.B., Wolfe R.R., Shenkin A. (2019). Hypoalbuminemia: pathogenesis and clinical significance. JPEN - J. Parenter. Enter. Nutr..

[bib75] Stares M., Swan A., Cumming K. (2021). Hypoalbuminaemia as a prognostic biomarker of first-line treatment resistance in metastatic non-small cell lung cancer. Front. Nutr..

[bib76] Sullivan P.Z., Niu T., Abinader J.F. (2022). Evolution of surgical treatment of metastatic spine tumors. J. Neuro Oncol..

[bib77] Suzuki H., Asakawa A., Amitani H., Nakamura N., Inui A. (2013). Cancer cachexia--pathophysiology and management. J. Gastroenterol..

[bib78] Switlyk M.D., Kongsgaard U., Skjeldal S. (2015). Prognostic factors in patients with symptomatic spinal metastases and normal neurological function. Clin. Oncol..

[bib79] Uei H., Tokuhashi Y. (2020). Prognostic scoring system for metastatic spine tumors derived from hepatocellular carcinoma. J. Orthop. Surg..

[bib80] Vincent J.L., Dubois M.J., Navickis R.J., Wilkes M.M. (2003). Hypoalbuminemia in acute illness: is there a rationale for intervention? A meta-analysis of cohort studies and controlled trials. Ann. Surg..

[bib81] Wilkes M.M., Navickis R.J. (2001). Patient survival after human albumin administration. A meta-analysis of randomized, controlled trials. Ann. Intern. Med..

[bib82] Wu J.M., Ho T.W., Lai I.R., Chen C.N., Lin M.T. (2021). Parenteral glutamine supplementation improves serum albumin values in surgical cancer patients. Clin. Nutr..

[bib83] Wu T.H., Tsai Y.T., Chen K.Y., Yap W.K., Luan C.W. (2023). Utility of high-sensitivity modified Glasgow prognostic score in cancer prognosis: a systemic review and meta-analysis. Int. J. Mol. Sci..

[bib84] Xu H., Han Z., Ma W., Zhu X., Shi J., Lin D. (2021). Perioperative albumin supplementation is associated with decreased risk of complications following microvascular head and neck reconstruction. J. Oral Maxillofac. Surg..

[bib85] Yang H., Wang K., Liang Z. (2020). Prognostic role of pre-treatment serum albumin in patients with nasopharyngeal carcinoma: a meta-analysis and systematic review. Clin. Otolaryngol..

[bib86] Yang M., Ma X., Wang P. (2022). Prediction of survival prognosis for spinal metastasis from cancer of unknown primary: derivation and validation of a nomogram model. Glob. Spine J..

[bib87] Yoshikawa N., Yoshihara M., Tamauchi S., Ikeda Y., Yokoi A., Kajiyama H. (2022). Hypoalbuminemia for the prediction of survival in patients with stage IVB cervical cancer. PLoS One.

[bib88] Zakaria H.M., Wilkinson B.M., Pennington Z. (2020). Sarcopenia as a prognostic factor for 90-day and overall mortality in patients undergoing spine surgery for metastatic tumors: a multicenter retrospective cohort study. Neurosurgery.

[bib89] Zang S., He Q., Bao Q., Shen Y., Zhang W. (2019). Establishment and validation of a novel survival prediction scoring algorithm for patients with non-small-cell lung cancer spinal metastasis. Int. J. Clin. Oncol..

